# Assessment of Chewing in Children With Down Syndrome

**DOI:** 10.1177/01454455221129992

**Published:** 2022-10-29

**Authors:** Chiara Ferrari, Elena Marinopoulou, Helena Lydon

**Affiliations:** 1 University of Galway, Galway, Ireland

**Keywords:** down syndrome, chewing assessment, chewing components, tongue lateralization, bite down, chew

## Abstract

In the present study a comprehensive protocol to assess chewing in four children with Down syndrome is provided and described. One baseline and four assessment meals were conducted across four textures of foods to investigate the presence or absence of components of chewing (bite down, chew and tongue lateralization), as well as movements associated with the development of chewing. Results showed that at baseline all participants ate their typical diet (i.e., pureed food) but no chewing components or movements were observed. The findings of the assessment protocol indicated that it offers a prescriptive assessment of chewing and its components across different food textures. The results of the assessment provided useful information for clinicians by identifying a potential starting point for interventions to address chewing deficits. Furthermore, the findings add to the existing literature on the role of tongue lateralization and specific tongue movements in chewing. Implications of the findings for chewing interventions and future research are discussed.

Chewing, also known as mastication, is the process that enables an individual to prepare food for swallowing so that it can be processed in the digestive system (Meenakshi & Paul, 2017). For most children, chewing efficiency is established over a 2-year time period in the absence of intervention (Wilson & Green, 2009). However, children with Down syndrome (DS) do not always follow this pattern and sometimes swallow food without chewing (Gisel et al., 1984). Lack of early experience with high-texture foods is an important variable which can contribute to chewing delays (Beckett et al., 2002), but children with DS also have anatomical and physiological differences in the mouth and throat areas which impact the development of oral motor skills such as feeding, cup drinking, chewing, and swallowing solid foods (Spender et al., 1996).

Anatomical differences in children with DS include small oral cavities with relatively large tongues and high palatal arches (Shapiro et al., 1967). These oral structural differences can negatively impact the occlusal of the jaw in which the teeth of both arches should fully interpose the teeth of the opposing arch (Farkas et al.,1985). To compensate for the malocclusion of the jaw, individuals with DS tend to either protrude the jaw to find more dental contact or interpose their tongue between the dental arches (English et al., 2002; Farkas et al.,1985). This potentially explains why children with DS tend to hold food in their mouths without chewing, also referred to as packing, while waiting for saliva to soften the food (Gisel et al., 1984). In addition, physiological differences in children with DS include low muscle tone, lax ligaments, and weak oral facial muscles, which can make jaw support difficult (Spender et al., 1996). Studies suggest that individuals with DS have slow mastication frequency and insufficient adaptation to increased food hardness, which consequently affects their ability to chew (Mazille et al., 2008).

Chewing is comprised of three main component skills: bite down, chew, and tongue lateralization. These components typically develop through three progressive stages starting from 6 months of age: vertical chewing, horizontal chewing, and rotary chewing. In the first stage, simple vertical jaw elevations are performed while the tongue also moves up and down (Bosma, 1986). This vertical movement of the jaw is referred to as “bite down.” The next stage in the development of chewing is characterized by the emergence of lateral jaw motion. At this stage, vertical movements are alternated to horizontal motions of the jaw and this is referred as “chew” (Arvedson et al., 1993). In the final stage of chewing development, the jaw moves in a rotary motion and at the same time, the tongue moves from side to side in order to manipulate the food into a bolus to prepare it for swallowing. This tongue movement is referred as “tongue lateralization.”

Wilson and Green (2009) reported that a distinct transition from vertical to rotary chewing in not supported in the literature, but rather that a more protracted trajectory occurs alongside changes in movement speed and the children’s sensitivity to bolus consistency. Woda et al. (2006) suggested that functional jaw control may develop earlier than tongue control, leading to the hypothesis that rotary chewing might not develop fully until the tongue is ready to support the jaw. In addition, they reported that adaptation of jaw control to different food consistencies was an important component in the development of rotary chewing. Phipps et al. (2022) suggested that the components involved with chewing develop only when children are exposed to higher texture foods.

It is unclear whether children with DS have problems with each component skill across textures. To date, literature has focused primarily on assessing chewing in adults with DS who can already perform a minimal level of chewing. For example, Allison et al. (2004), assessed chewing in adults with DS by taking data on masticatory time (MT), number of masticatory cycles (MC), and number of open masticatory cycles (OMC) across four different foods of varying hardness: pureed, mashed, bite sized, and regular. Results showed that MT and MC were positively correlated with the hardness of the food eaten. However, children with DS often eat only low textured food and when assessing chewing, MC and OMC would fail to provide useful data on the three main chewing components: bite down, chew, and tongue lateralization.

In recent years, a team of physiotherapists developed The Karaduman Chewing Performance Scale (KCPS) to determine via observation the overall level of chewing function in children with neuromuscular diseases (Serel Arslan et al., 2016). The authors operationally defined chewing as a predictable sequence of movements from the first step of accepting food within the mouth to the final step of grinding and softening the food via a series of MC (Serel Arslan et al., 2018). The scale was found to be a reliable and valid functional instrument to measure chewing (Serel Arslan et al., 2018). However, the KCPS does not provide a clear level of functioning of the separate chewing components which could better guide intervention. In this regard, Phipps et al. (2022) assessed tongue lateralization with and without food present in the mouth. This method of assessment allowed for the observation of tongue movements (left, right, as well as movements to the lower and upper lip) required to perform tongue lateralization. To date, no research has assessed the movements associated with the other chewing components: bite down and chew. The development of an assessment method which allows for more specific information on the movements associated with all three chewing components could provide: (a) important information on the level of functioning of each individual chewing component, and (b) specify behavior analytic interventions to teach specific chewing components as needed.

The present study provided a component-composite protocol to assess chewing in children with DS. This study separately assessed the level of functioning of each chewing component (bite down, chew, and tongue lateralization) across four different textures of food. In addition, bite and chew movements were assessed in order to identify if the children presented with the prerequisites to preform the chewing components. Simialry, tongue movements (left, right, up, and down) were assessed as precursors for the development of tongue lateralization. The KCPS was used to assign an overall level of chewing function to each participant and MT, MC, and OMC were also recorded. Finally, this study assessed mealtime behaviors exhibited by the participants to better understand the variables maintaining chewing.

## Method

### Recruitment and Inclusion/Exclusion Criteria

Families were recruited through social media and local organizations who provided services to children with intellectual and developmental disabilities. Participants were four children with a diagnosis of DS and their parents. As parents completed the assessment in their own homes, at least one parent was required to be involved in all phases of the assessment. Inclusion criteria included children: (a) with a diagnosis of DS, (b) between 3 and 10 years of age, and (c) who ate only low textured foods due to their inability to chew. Children with the inability to swallow foods were excluded, as determined by their general practitioner (GP). For privacy, each participant was given a pseudonym.

### Measures

An initial evaluation in the form of interview was conducted with the children’s parents and they completed the *Karaduman Chewing Performance Scale***(**KCPS) and a food inventory.

#### Initial evaluation form

Williams and Foxx (2007) developed the initial evaluation form, a parent interview which was delivered prior to the assessment. Information such as participants’ medical history, feeding history and any behaviors exhibited during typical mealtimes were recorded.

#### Karaduman Chewing Performance Scale (KCPS)

The Karaduman Chewing Performance Scale is used to determine the overall level of chewing exhibited by each participant (Serel Arslan et al., 2016). The scale classifies chewing performance into five levels (with each ranging from 0 to 4) based on the skills performed by the child during an observation. The scale provides an explanation of each level to differentiate scoring. See Table 1 for more details. Level 0 is normal chewing function, Level 1 is the child chews, but there are some difficulties in transition from food to bolus, Level 2 is the child starts to chew, but he/she cannot hold the food in the molar area, Level 3 is the child bites but cannot chew, and Level 4 is the child cannot bite and chew. The KCPS has been found to have high reliability .93, as well as good test-retest reliability .83 (Serel Arslan et al., 2018).

**Table 1.** The Karaduman Chewing Performance Scale.

The steps of scale (0–4)	Explanation
0: Normal chewing function	Child can hold and bite the solid food
	Child can transfer the solid food with lateral tongue movements to the molar area
	The food can be broken down between (pre)molar teeth into small pieces with the lateral and rotational tongue movements
	The bolus formation after chewing is transferred to oropharynx with elevation and retraction of the tongue and then swallowed
1: The child chews, but there are some difficulties in transition food to bolus	Child can hold and bite the solid food
	Child can transfer the solid food with lateral tongue movements to the molar area
	There is an inefficacy in breaking down the food between (pre)molar teeth into small pieces with the lateral and rotational tongue movements
	The food which cannot be broken down efficiently is transferred to oropharynx with elevation and retraction of the tongue and then swallowed
2: The child starts to chew, but he/she cannot hold the food in the molar area	Child can hold and bite the solid food
	Child can transfer the solid food with lateral tongue movements to the molar area
	The food cannot be hold in the molar area due to the problem in lateral and rotational movements of the tongue
	The food can not broken down into small pieces efficiently
	There is a problem about turning the food into bolus formation
	The food is either transferred to oropharynx with elevation and retraction of the tongue or throwed out of the mouth
3: The child bites but cannot chew	Child can hold and bite the solid food
	Child cannot manage the other necessary steps for chewing
4: The child cannot bite and chew	There are problems in all steps of chewing

#### Food inventory

A food inventory was used to indicate foods which the child ate from the following food groups: protein, starch, fruit, and vegetables (Williams & Foxx, 2007). The inventory was provided to the parents who endorsed each food their child consumed with a tick and identified each food their child did not eat with an X.

#### Participants

Liam was a 7 year-old boy with DS and sleep apnea. He attended a special school and he used sign language to communicate. His parents reported that he could self feed, had good fine and gross motor skills, but could not chew. In addition, his daily diet consisted of a narrow range of foods (mashed beef mixed with potatoes and carrots and stewed fruits) and he refused any novel foods.

Thomas was a 10 year-old boy with DS. He attended a mainstream school and engaged in vocal communication in full sentences. His parents reported that he presented with food selectivity, which they believe began when he started gagging on foods due to his inability to chew. Thomas only ate mashed food and refused all other textures. His diet consisted of a narrow range of foods including banana, Weetabix, chicken and mushroom pasta (a specific brand), spaghetti hoops, tomato soup, custard and yogurt, and he refused any novel foods.

Laura was a 5 year-old girl with DS, intellectual disability, and brain injury. Laura had multiple medical and behavioral issues since birth. She was enrolled in a special school and had limited expressive language. Laura previously attended a Messy Food Clinic at a hospital for a few years but did not progress to textures. Her parents reported that in addition to the inability to chew, Laura packed food in her mouth and cheeks for long times during meals.

Eric was a 5 year-old boy with DS and infantile spasms. He was enrolled in a special school and had limited expressive language. His parents reported that in addition to the inability to chew, at the age of two, Eric developed uncontrolled infantile spasm for over 2 years. During this time, Eric lost all the skills he learned, including finger feeding himself and sucking from a straw. His parents also reported that at the time of the assessment, Eric had only recently started to put things in his mouth.

Parents gave written consent for their children to participate in the study, and for their children to be video recorded as part of the study.

### Design

A series of case studies were undertaken to examine participants chewing behavoiurs when presented with the typical food (baseline meal) and when the texture of target foods was increased.

### Settings and Foods

The present study consisted of five meals (one baseline and four assessment meals) conducted by the participants’parents one meal per day across five consecutive days. Baseline and assessment meals took place within the child’s home, at the kitchen table with the child seated on a chair and the parent presenting the food. The kitchen was equipped with table, chairs, kitchen appliances, and a video camera to record the meals. Five target foods were chosen by the parents and these were foods already present in the children’s diet (see Table 2). Exceptions to this were made for Liam and Thomas. For Thomas, banana was used twice due to Thomas’ narrow range of foods and the fact that tomato soup and custard could not be prepared in line with the different consistencies required for the assessment. For Liam, five target foods were initially chosen for the assessment. However, following Meal 1, Liam’s mum reported that he was very distressed, and thus, the remainder of the assessment was completed using Liam’s typical daily meal (beef mixed with potatoes and carrots). The consistency of the meal was altered (i.e., presented as pureed, minced and moist, soft and bite-sized, and regular) in order to meet the different textures of the assessment.

**Table 2.** Target Foods per Each Participant Across Baseline and Assessment Phases.

Participant	Baseline (texture)	Phase 1 and 3	Phase 2
Liam	Meat mixed with potatoes and carrots (puree)	Beef mixed with potatoes and carrots	Yogurt
Thomas	Cow and gate pasta (puree)	Spaghetti hoops	Yogurt
		Cow and gate pasta	
		Weetabix	
		Banana	
		Banana*	
Laura	Egg and Beans (puree)	Chicken	Yogurt
		Carrots	
		Broccoli	
		Cauliflower	
		Potato	
Eric	Porridge	Potato	Yogurt
		Carrot	
		Chicken	
		Broccoli	
		Cauliflower	

* Food used twice due to narrow diet and the need to prepare all target foods at each texture.

#### Textures

During the baseline meal all foods were presented by the parent, in the texture typically consumed by their child. All four particpants currently consumed only pureed food. Each target food presented during the assessment meals was manipulated to create four food textures: (a) pureed, (b) minced and moist, (c) soft and bite-sized, (d) regular. The four textures were prepared in accordance with the International Dysphagia Diet Standardization Initiative (IDDSI). The IDDSI has created global standardized definitions for texture-modified diets and thickened liquids to improve the safety and care for individuals with swallowing difficulties, also referred as dysphagia. The IDDSI framework consists of a continuum of eight levels (0–7), in which drinks are measured from Levels 0 to 4, while foods are measured from Levels 3 to 7. IDDSI tests are intended to confirm the textural characteristics of a particular product at the time of testing. The IDDSI has been shown to have strong consensual and criterion validity (Steele et al., 2018). The IDDSI can be used reliably by clinicians to capture diet texture restriction and progression in people with dysphagia (Steele et al., 2018) and to guide health care facilities to provide food to at-risk patients (Wu et al., 2022).

Although none of the participants in this study had swallowing difficulties, their inability to chew put them at risk for choking, and therefore, we controled for the food texture across the assessment meals. Each texture was tested through the use of testing methods for texture modified foods recommended by the International Dysphagia Diet Standardization Initiative (IDDSI): (a) fork drip test, (b) fork pressure test and spoon pressure test, (c) chopstick test, and (d) finger test.

### Videos and Data Collection

Video recordings were taken by the participants’ parents and data were coded by the first author while watching the recorded videos. Parents were instructed to take video recordings with a full-face camera view, placed at 1.2 m from the participant. The frame was delimited 10 cm beyond the shoulders and 10 cm from the top of the head of the participant.

### Dependent Variables

Data were coded on the following behaviors: bite, chew, tongue lateralization, tongue lateralization with a bite of food, and mouth clean. *Bite* (B) was defined as each time the child’s teeth and jaw completed one single up-and-down vertical motion such that the upper and lower teeth made contact to break food down in the mouth. *Chew* (C) was defined as three vertical jaw motions within 15 seconds of the food entering the mouth. If the researcher did not observe the food on the teeth (e.g., lips were closed) but observed chewed food in the mouth (a product of chewing), the researcher recorded an occurrence of chew. *Tongue lateralization* (TL) was defined as each tongue movement from side to side, past the vertical center of the mouth. If the researcher did not directly observe tongue lateralization, the researcher recorded non-observed. *Tongue lateralization with a bite of food* (TLB) was defined as each time the child’s tongue moved the bite of food to the teeth to chew it. *Mouth clean* (MoC) was defined as no food or drink larger than a pea visible before the next bite or 30 seconds after acceptance.

Data were also coded on bite, chew and tongue movements. *Bite Movement* (BM) was defined as the single up-and-down vertical motion such that the upper and lower teeth make contact without food present in the mouth. *Chew movement* (CM) was defined as three vertical jaw motions within 15 seconds without food present in the mouth. *Lateral Tongue Movement* (LTM) was defined as every time the tongue moved on either corner of the mouth in a lateral motion. *Down Tongue Movement* (DTM) was defined as every time the child stuck the tongue out on lower side of the mouth. *Up Tongue Movement* (UTM) was defined as every time the child stuck the tongue out on the upper side of the mouth.

Data were also coded on acceptance, refusal, expulsion, disruptive behavior, and gagging. These behaviors were considered to be secondary to the primary goal of chewing, but were recorded because they are often an important aspect of feeding programs. *Acceptance* (A) was defined as consuming the target food within 30 seconds. *Refusal* (R) was defined as not accepting the target food, within 30 seconds of being presented with a bite. *Expulsion* (E) was defined as food appearing past the border of the lips after food had been accepted in the mouth. *Disruptive Behavior* (D) was defined as problem behaviors that interfered with the meal and included leaving their seat, sliding down in their chair, pushing the food away or negative vocalizations (shouting, screaming, crying). Gagging (G) was defined as the child making a gagging sound or indications (neck extension, tongue protrusion, changes in skin color) of gagging. Other dependent variables under investigation in this study included masticatory time, masticatory cycles, open masticatory cycles, and packing. *Masticatory Time (MT)* was defined as the number of seconds between the moment food is placed in the mouth and swallowing, identified by the immediate swallow after the end of rhythmic rotary movements. *Masticatory cycles (MC)* was defined as the number of masticatory actions during the MT period, which included all the rotary patterns, with and without lip closure. *Open masticatory cycles* (OMC) was defined as the number of masticatory actions taken with separated lips during the MT period. *Packing* (P) was defined as any food, that remained in the child’s mouth 30 s after the acceptance of a bite.

### Parent Training

Prior to beginning the baseline and assessment meals, the researcher trained the participants’ parents, via telehealth, on the steps necessary to conduct the assessment in their own homes. The researcher provided instruction, modeling of the three phases of the assessment (i.e., tongue lateralization and chewing, biting and chewing, and tongue lateralization), rehearsal and feedback. Parents received a manual with instructions on how to take the video recordings, and on how to determine and ensure the correct texture of food based on the IDDSI recommendations. Prior to beginning the assessment, parents completed a role play with the researcher. Parents demonstrated how they would set up the camera, prepare each of the textures, and present the three phases of the assessment. The researcher provided feedback on their performance prior to the start of the assessment (i.e., feedback on the way the camera was set up, whether the food was prepared and cut in the correct way and presented in the correct order according to the phases of the assessment). Finally, following the completion of each meal session, the researcher reviewed the videos of the meal and called the participant’s parents for 30 to 45 minutes to review the assessment and address questions or concerns. At this time the researcher also addressed any procedural issues observed during the videos. Parents received daily feedback and support throughout the five assessment days.

### Baseline and Assessment Procedure

#### Baseline meal

During the baseline meal all 10 bites of food were presented by the parent, in the texture typically consumed by their child (i.e., puree). The child was seated in a chair at the kitchen table. All bites were presented in the middle of the mouth with the instruction “Take a bite.” Bites were presented every 30 seconds. No consequences were delivered to the child upon acceptance or refusal of the bite of food. Mastication time was recorded for each bite (i.e., the number of seconds between the moment food is placed in the mouth and swallowing). The purpose of the baseline meal was to gather data on the participants’ mealtime behaviors, their average duration to swallow a bite of food, and to assess which component skills or movements were present during a typical meal. The inclusion of the baseline (typical meal) and the purred meal may both be useful components within the assessment even though foods are presented at the same texture as the inclusion of the pureed meal may help the children familiarize with the assessment protocol. Data were taken on all dependent variables.

#### Assessment meals

The five target foods chosen for each participant were presented at each meal, therefore, each meal consisted of 10 bites of food and two dabs of yogurt placed at the corner of the child’s mouth. Each assessment meal involved three phases: (1) tongue lateralization and chewing assessment (five bites, one bite of each target food), (2) biting and chewing assessment (five bites, one bite of each target food), and (3) tongue lateralization assessment. Each of the 10 bites were presented for up to 30 seconds in duration, therefore, each meal lasted up to 6-minutes. During the four assessment meals, the five target foods for each participant were presented as pureed, minced and moist texture, soft and bite-sized and in regular texture. The texture of the food was increased across meals, following the presentation of 10 bites at the previous texture.

#### Tongue lateralization and chewing assessment (Phase 1)

During Phase 1, the parent presented a spoonful of each target food in the middle of the mouth with the instruction “Take a bite.” The aim of this phase was to establish if the child could engage in tongue lateralization to reposition the food to the molars and chew the food. No consequences were delivered to the child for acceptance or refusal of the target food and after 30 seconds the parent moved to to the next target food in rotation. Data were taken on all dependent variables.

#### Tongue lateralization assessment (Phase 2)

In Phase 2, the parent placed some yogurt on the corners (left and right) of the child’s mouth and they instructed the child to “lick the yogurt off.” The objective of this phase was to examine if the child could engage in tongue movements associated with chewing. No consequences were delivered to the child for correct or incorrect responses. Data were taken on TL, LTM, DTM, UTM, and on the challenging behaviors exhibited during the phase.

#### Biting and chewing assessment (Phase 3)

During Phase 3, the parent first modeled the bite down movement and then presented a spoonful of each target food to the side of the child’s mouth in order to be positioned on the molar teeth with the instruction “bite down.” The aim of this phase was to investigate if the child could engage in bite or chew behavior with the target food if the food was positioned at the side of the child’s mouth. No consequences were delivered to the child upon acceptance or refusal of the target food and after 30 seconds parents moved to the next food in rotation. Data were taken on all dependent variables.

### Interobserver Agreement and Procedural Fidelity

All video recorded meals were observed in a random order and coded for all dependent variables by both the researcher (first author) and seperately by an independent observer (second author). IOA was conducted for 100% of all trials conducted with Liam, 80% of trials with Thomas, 100% of trials for Laura and 80% of trials for Eric. Inter-observer agreement was calculated by dividing the total number of agreements by the sum of agreements plus disagreements and converting the result to a percentage. An agreement for bites accepted was defined as both observers marking the trial the same way (i.e., by marking ‘A’ to indicate the participant accepted the target food within 30 seconds). A disagreement was defined as both observers not marking the trail the same way (i.e., one marking A to indicate the participant accepted the target food within 30 seconds and the other observer marking R to indicate refusal of the target food).

The mean IOA for all four participants was 88.9%, ranging from 76.5% to 97% agreement. IOA was highest for Liam and Eric at 95% and 97% agreement respectively. The lowest level of agreement was recorded for Laura at 76.5% and Thomas was 87% agreement.

Fidelity checks were conducted for 100% of all trials conducted by the parent of the participant. Correct implemetation of the assessment procedure was defined as parents accurately implemetning each phase of the assessment procedure. A phase was defined as correct if parents presented (i) food of the correct texture, (ii) the correct verbal antecedent, and (iii) provided no consequences for acceptance or refusal.

Parents accuracy of implementation of the assessment procedure was recorded throughout the intervention and was calcualted by dividing the number of correctly implemented phases by the total number of phases on which fidelity was checked for each parent.

Fidelity scores averaged 93.65% and ranged from 90.8% to 96.6% across parents and meals. Liam’s parent displayed levels of fidelity ranging from 80% to 100% across 4 days, Thomas’s parent displayed levels of fidelity ranging from 91% to 100%, Laura’s parent displayed levels ranging from 83% to 100% across 4 days and Eric’s parent displayed levels ranging from 84% to 100%.

## Results

### Chewing Components and Mouth Clean

**Figure 1.**
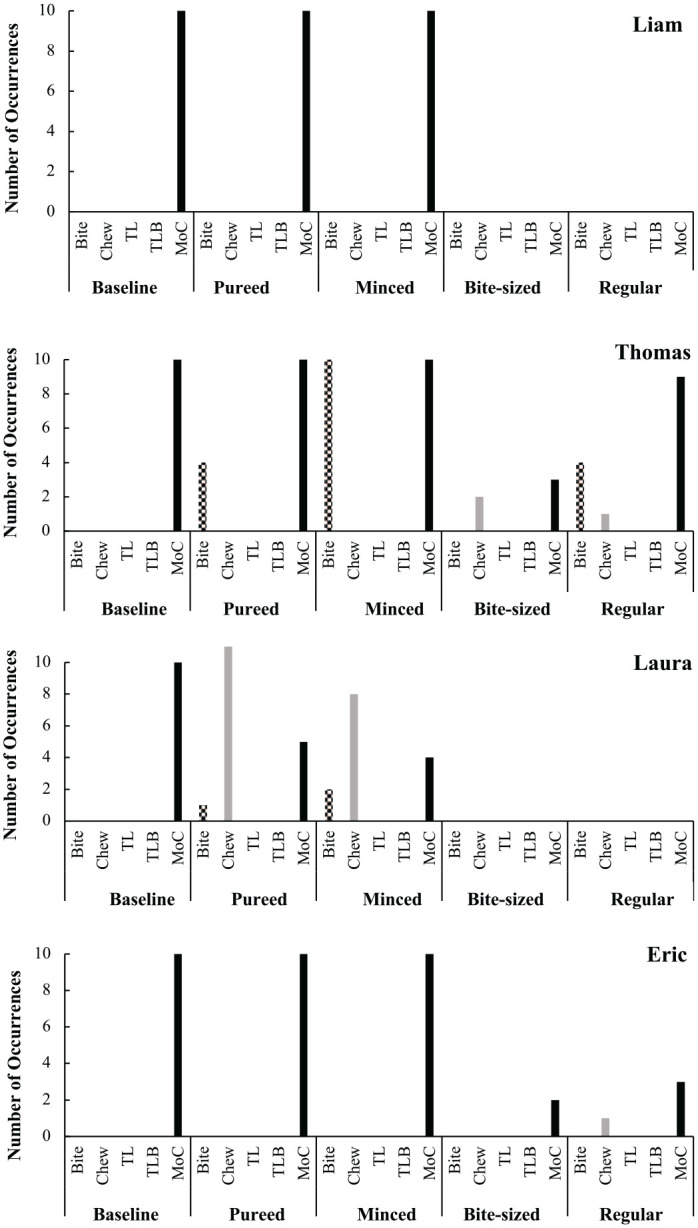
The number of occurrences of the components of chewing and mouth clean per each participant across the assessment. *Note.* TL = tongue lateralization; TLB = tongue lateralization with bite; MoC = mouth clean.

At baseline, when the food was pureed all four participants were presented with and consumed 10 bites (100% mouth clean) of their typical foods. None of the four participants displayed bite down, chew, tongue lateralization or tongue lateralization with bite during baseline (see Figure 1). The results showed that when presented with the five target foods at pureed and minced and moist textures all participants engaged in more of the components of chewing and mouth clean, and less challenging behavior.

During assessment meals, when the food was pureed or minced and moist, it was noted that three of the four participants maintained high levels of mouth clean (see Figure 1). Thomas also began to display bite behavior, when presented with either pureed, minced and moist textures. Laura engaged in lower levels of mouth clean during pureed and minced and moist textures, but also began to display bite and chew. In contract, when the food was soft and bite-sized or regular, all four participants showed a reduction in mouth clean from previous meals. In addition, Thomas showed lower levels of bite than in pureed or minced and moist, but did display some chew behavoiurs.

### Bite, Chew, and Tongue Movements

**Figure 2.**
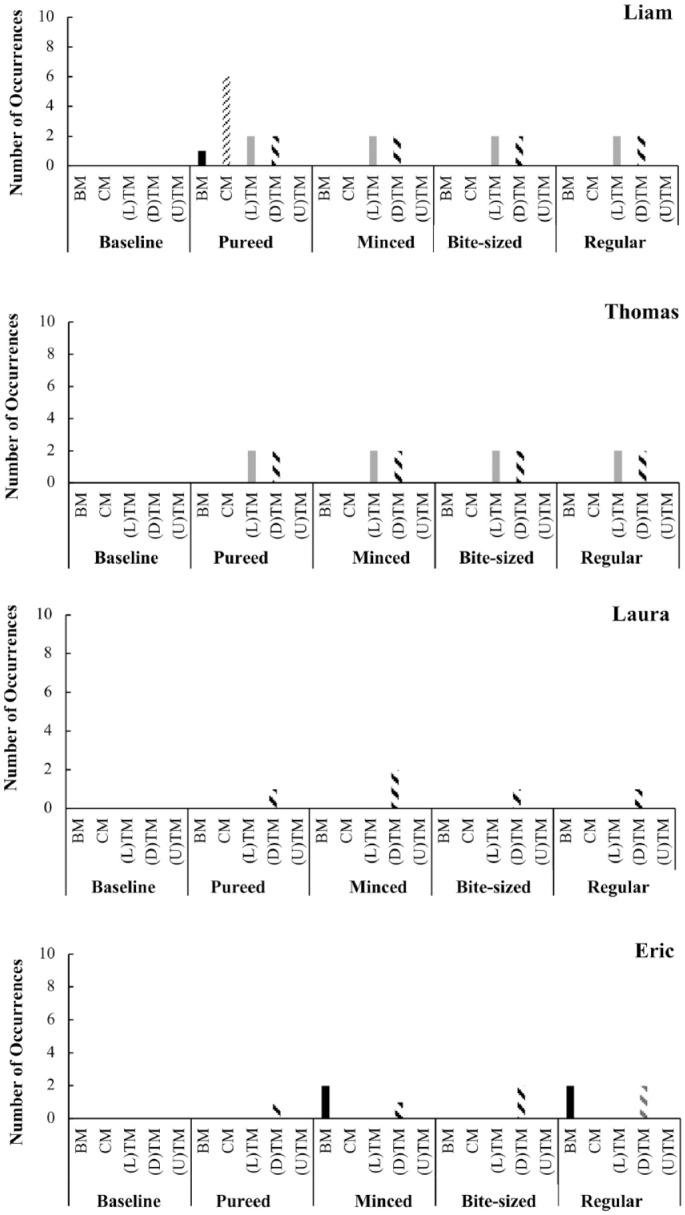
The number of occurrences of bite, chew and tongue movements per each participant across the assessment. *Note*. BM = bite movement; CM = chew movement; (L)TM = lateral tongue movement; (D)TM = down tongue movement; (U)TM = up tongue movement.

At baseline, when the food was pureed, no occurrences of bite movement, chew movement or tongue movements were observed for any participants (see Figure 2).

During the assessment meals, all four participants were more likely to display some of the component movements. Down tongue movement was evident for all four participants, and lateral tongue movement was displayed by two of the four participants. In addition to tongue movements, when the food was pureed Liam displayed one bite movement and eight chew movements, and Eric displayed two bite down movements when the food was minced and moist and two bite down movements when the food was regular. For all children, no UTM was observed throughout the assessment.

### Acceptance and Challenging Behavior

**Figure 3.**
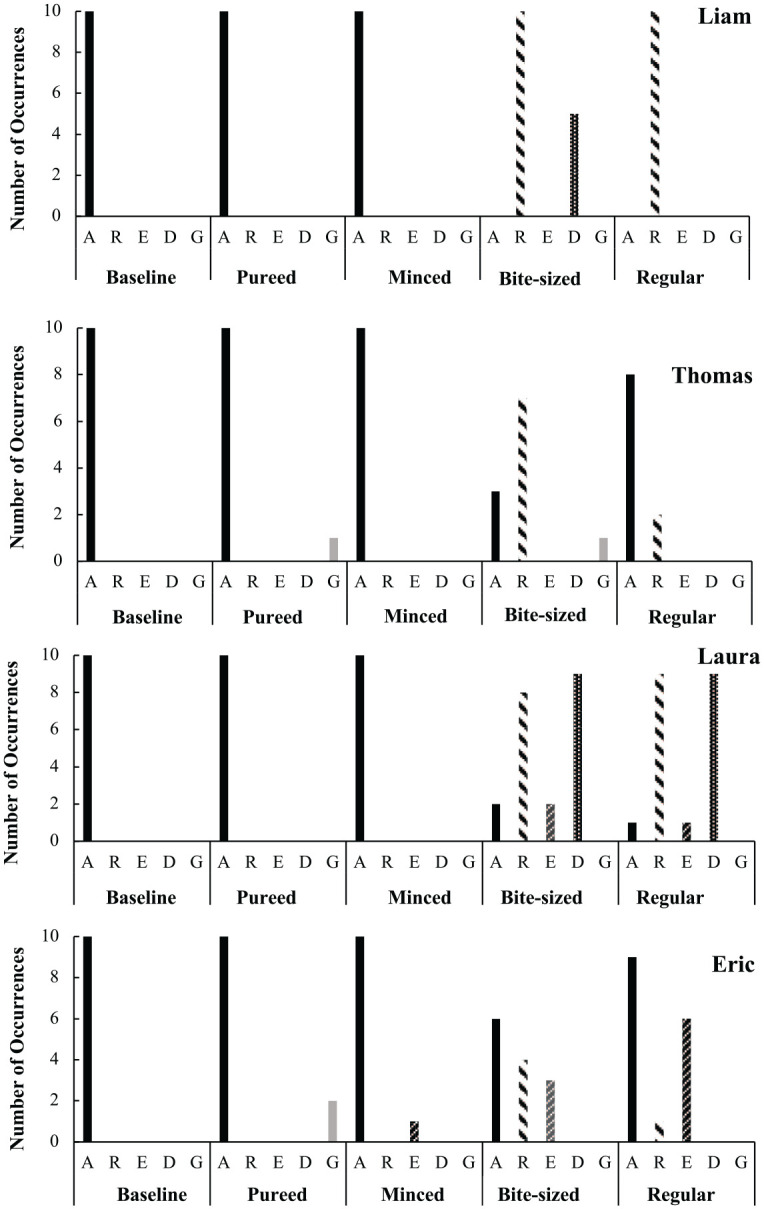
Number of occurrences of acceptance, refusal, disruption, expulsion and gagging for each participant across baseline and assessment meals. *Note*. A = acceptance; R = refusal; E = expulsion; D = disruption; G = gagging.

At baseline, when the food was pureed there was high acceptance (100%) for all four participants and no occurrences of refusal, expulsion, gagging or disruptive behavior were observed (see Figure 3).

When the foods were pureed or minced and moist, no occurrences of refusal or disruptive behavior were observed across all four participants. Low levels of gagging were noted for Thomas and Eric in the pureed meal. Similary, low levels of expulsion were noted for Eric during the minced and moist meal. In contrast, when the texture of the food increased to soft and bite-sized or regular, an increase in refusal was observed for all four participants. Refusal was accompanied by disruptive behavior, explusion, or both, for three of the four participants (Liam, Laura, and Eric).

### Masticatory Time, Masticatory Cycles, Percentage of Packing, and KCPS Level of Chewing

At baseline, Liam ate 10 bites (100% mouth clean) in 1 minute and 52 seconds with an average of 2.6 seconds per bite of food (*SD* = 0.5). During the assessment meals his average MT increased from 2.3 seconds in the pureed meal (*SD* = 0.4) to 2.6 seconds with the minced and moist meal (*SD* = 0.5). MT was not calcualted for soft and bite-sized or regular meals as no bites were consumed. No MC, OMC or packing were observed throughout the assessment and level 4 of KCPS was assigned to Liam’s level of chewing (see Table 3).

**Table 3.** Masticatory Time and Percentage of Packing, Masticatory Cycles, per Meal and Particpant.

Participant	Texture	MT-Min	MT-Max	MT-Mean (SD)	Packing (%)	MC	OMC	KCPS level
Liam	Baseline	2 seconds	3 seconds	2.6 (0.5)	0	0	0	
	Pureed	1 seconds	3 seconds	2.3 (0.4)	0	0	0	
	Minced	2 seconds	5 seconds	2.6 (0.5)	0	0	0	4
	Bite-sized	/	/	/	/	/	/	
	Regular	/	/	/	/	/	/	
Thomas	Baseline	1 seconds	2 seconds	1.2 (0.2)	0	0	0	
	Pureed	1 seconds	2 seconds	1.5 (0.5)	0	0	0	
	Minced	1 seconds	3 seconds	2 (1.0)	0	0	0	4
	Bite-sized	3 seconds	4 seconds	3.7 (0.6)	0	0	0	
	Regular	1 seconds	4 seconds	2.22 (0.7)	0	0	0	
Laura	Baseline	7 seconds	35 seconds	23.2 (21.6)				
	Pureed	6 seconds	25 seconds	18.7 (11)	70	0	0	
	Minced	10 seconds	20 seconds	12.5 (5)	60	0	0	4
	Bite-sized	/	/	/	/	/	/	
	Regular	/	/	/	/	/	/	
Eric	Baseline	4 seconds	4 seconds	4 (0)				
	Pureed	6 seconds	20 seconds	10.12 (4.5)	10	0	0	
	Minced	6 seconds	30 seconds	10 (7.7)	0	0	0	4
	Bite-sized	6 seconds	6 seconds	6 (0)	10	0	0	
	Regular	10 seconds	10 seconds	10 (0)	10	0	0	

At baseline, Thomas consumed 10 bites (100%) in 1 minute and 30 seconds with an average of 1.2 seconds per bite of food (SD = 0.2). During assessment, the average MT per bite of food increased from 1.5 seconds in the pureed meal (SD = 0.5), to 2 seconds in the minced and moist meal (*SD* = 1.05) and to 3.7 seconds when the texture increased to soft and bite-sized (*SD* = 0.6). During the regular meal, the average MT decreased to 2.22 seconds per bite of food (*SD* = 0.7). No MC, OMC or packing were observed throughout the assessment meals. Level 4 of KCPS was assigned to Thomas’s level of chewing.

At baseline, Laura consumed 10 bites (100%) in 5 minutes and 54 seconds with an average of 23.22 seconds per bite of food (*SD* = 21.64). During assessment the average MT decreased from 18.66 seconds in the pureed meal (*SD* = 11) to 12.5 seconds in the minced and moist meal (*SD* = 5). Similar to Liam, MT was not calcualted for soft and bite sized or the regular meal as no bites were consumed. Laura packed seven bites (70%) during the pureed meal and six bites in the minced and moist meal (60%). No MC or OMC were observed throughout the assessment meals and level 4 of KCPS was assigned to Laura’s level of chewing.

At baseline, Eric consumed 10 bites (100%) in 1 minute and 20 seconds with an average of 4 seconds per bite of food (*SD* = 0). During assessment the average MT decreased from 10.12 seconds during the pureed meal (*SD* = 4.55) to 10 seconds during the minced and moist meal (*SD* = 7.74). Average MT increased from 6 seconds in the soft and bite-sized meal (*M* = 6, SD = 0) to 10 seconds in the regular meal (*M* = 10, *SD* = 0). Eric packed one bite (10%) in the pureed meal, one bite in the minced and moist meal (10%) and one bite in the soft and bite-sized meal (10%). No MC or OMC were observed throughout the assessment meals and level 4 of KCPS was assigned to Eric’s level of chewing.

## Discussion

The findings of the current study add to the existing literature on chewing in two primary areas. The present study provides a protocol to assess chewing and its components across different food textures with children with DS thus providing information for determining a starting point for chewing intervention. The findings also add to the existing literature on the role of tongue lateralization and specific tongue movements in chewing. If replication of these results occur, the current findings suggest a possible link between LTM and packing, and provide preliminary evidence for the sequence of development of tongue movements for children with DS.

The findings of the current assessment indicated that the three phases of the assessment protocol were necessary in gathering information about each child’s ability to perform the components of chewing, the movements associated with chewing and disruptive mealtime behaviors. This protocol addresses chewing as a composite skill that can be broken down into component skills and movements to provide greater detail on a child’s ability to chew. Current assessment tool such as the KCPS may be useful in identifying whether the steps occurred in the right sequence, but when assessing children with no current ability to chew, the scale does not provide sufficient information. For example, in the current study, the KCPS indicated that all participants presented as having the same level of chewing while the present protocol allowed the researcher to identify in more detail the different skills present for each child. Thus, the primary benefit of the present protocol is that it not only enables clinicians to gather information regarding chewing as a sequence, it also differentiates the presence or absence of the component skills and movements necessary to develop chewing. This enables clinicans to identify the most appropriate starting point for an intervention.

Results of the assessment protocol showed that all children ate their typical food but none utilized the components of chewing during their typical meals. This finding is problematic as existing research indicates that a lack of appropriate early experience with high-texture foods contributes to delays in the development of chewing, thus, emphasizing the critical role of practice in the development of rotary chewing (Beckett et al., 2002). Similarly, Phipps et al. (2022) also advocated for practice with increasingly higher texture foods in order to develop the basic skills necessary to consume regular texture foods.

High incidents of mouth clean were noted for all four participants in the first two assessment meals when the texture was pureed and minced and moist. Two participants swallowed the foods presented to them without displaying any of the chewing components. This finding may be accounted for by the fact that rotary chewing is required for hard foods, while simple contact between tongue and palate is sufficient for soft foods (Allison et al., 2004). However, two of the participants did begin to display bite and chew during the first two assessment meals. These findings are noteworthy as they indicate that some children with DS can perform some of the skills needed for chewing at a slightly higher texture to their typical meals, and thus this may be the most appropriate starting point for intervention.

As the participants progressed to the higher textures (soft and bite-sized and regular), there was a reduction in the number of bites consumed across all participants, and a decrease in the component skills. These data suggest that participants may not have consumed the foods as they did not have the skills necessary to manipulate the food appropriately in order to consume it. However, there were also some exceptions as one participant (Thomas) ate three bites in the minced and moist meal and eight bites in the regular meal. For Thomas, this could be explained by the soft foods chosen for the assessment due to his food selectivity. The foods chosen for Thomas (banana, spaghetti hoops, cow and gate pasta and Weetabix) were all foods that could melt in his mouth without requiring him to chew. In addition, for Thomas all bite downs and chews occurred with a potrusion of the jaw and the tongue partially placed in between the teeth. This is a behavior frequently found in children with DS as a result of a malocclusion of the jaw and lack of jaw control (Farkas et al., 1985).

While two of the participants (Thomas and Laura) displayed some bite down and chew behaviors across the assessment meals it must be noted that they were not performed as part of a chewing sequence and not with higher texture food. In addition, the low rates (of bite down and chew) recorded suggested that they were not fluent in performing the two component skills. As previous research suggests, jaw control to different food consistencies is an important component in the development of rotary chewing (Wilson & Green, 2009). Although these components are necessary to develop rotary chewing, other components, such as tongue lateralization may be necessary to develop the skill.

The current research also assessed the basic skills (i.e., movements) required to perform each chewing component. Evaluation of these behaviors enabled the researchers to identify whether the children possessed the prerequisite skills (i.e., oral motor movements) necessary to perform the chewing components. At baseline, these findings mirrored the results obtained for the component skills as none of the participants displayed the movements necessary (i.e., bite movement, chew movement or tongue movement) thus emphasizing the lack of opportunities to practice these skills during their typical meals.

During the assessment meals, bite movement and chew movements were observed for the two participants who did not display any chewing components (Liam and Eric). These findings emphazied that higher texture foods encourage greater tongue movement and thus highlighting the need to continue to present these textures to children in order to support them to practice the skills necessary to develop chewing.

Results of the current assessment also showed that during the assessment meals, there were greater instances of DTM for all four participants and LTM for two of the four participants. As DTM was evident for all children within the current study, and UTM was not observed for any of the participants it may suggest that DTM emerges first for children with DS. However, if the children only perform DTM this may impact on their ability to manipulate food in their mouth prior to swallowing. In addition, as none of the children engaged in UTM, this may indicate that this skill is more challenging to preform and may require specific practice in order to develop this skills for children with DS.

Furthermore, LTM was only preformed by two chidlen during the assessment meals. It is noteworthy that the two children who did not preform any LTM across all four assessment meals were noted to engage in packing, indicating that the absence of LTM and poor tongue mobility may be associated with packing. These results may suggest that tongue lateralization requires the development of different tongue movements (down, up, left, and right) prior to reaching fluency as a chewing component itself. These findings align with previous research, which suggests that rotary chewing might not develop fully until the tongue is ready to support the jaw (Woda et al., 2006).

Existing literature suggest that behavioral issues exhibited by children with DS in combination with physical and medical issues can negatively impact feeding (Pipes & Holm, 1980). Within the current study, an inverse relationship between acceptance and challenging behaviors was observed for all participants and differences between the behaviors exhibited were found. This finding highlighted that in order to continue to gain acceptance during a intervention for chewing it may be best to target the most appropriate texture (i.e., one texture above their typical meal) based on their current skill levels, as higher textured foods result in the occurrence of challenging behavior.

The current findings reported that MT increased across textures for two of the participants. Data for the two remaining particpants were not possible to interpret in this way due to the high rates of refusal during the higher texture meals (soft and bite-sized and regular). This finding is in line with previous literature which reports that MT and MC increased from pureed to regular texture (Hennequin et al., 2005). Data on MT and MC should continue to be gathered, in particular during interventions for chewing, as they may provide valuable information on the fluent preformance of each of the compent skills.

### Limitations and Future Directions

Although the present study addresses a novel area of chewing, this study has also several limitations. First, the target foods chosen for the assessment were foods already present in the children’s diet. However, for two of the participants this was not possible due to the limited range of foods within their current diet. Thus, the foods chosen for Thomas were manipulated to create four textures but they did not meet the IDDSI reccomandations for soft and bite-sized and regular textures, which may have impacted on his acceptance of food in those meals. For Eric, only one food was chosen for the assessment and it was presented in four different textures according to the IDDSI. Future research could exclude participants who also score high for food selectivity as this issue may need to be addressed seperately to their chewing deficit. Secondly, although modeling of the bite down movement was successful in testing bite down in three participants, a limitation of this study is that imitation skills were not assessed prior to the beginning of the assessment. Finally, for one participant (Laura), the presence of the video camera during the assessment was very distracting which may have impacted on her responding during assessment meals.

Future research should investigate whether reaching fluency of bite down, chew and tongue lateralization is enough to develop chewing or if the behaviors need to be taught as part of a sequence of behaviors. In addition, future research should investigate whether targeting each of the chewing components simultaneously is more effective than teaching each of the skills individually in sequence. Furthermore, future research could examine if reaching fluency of the chewing component movements in the absence of food would help develop the component skills of bite down and chew required to develop rotary chewing.

In addition, different tongue movements may be prerequisites for tongue lateralization. The findings of the current study provided preliminary evidence of the developmental sequence of tongue movements for children with DS, as all children displayed the ability to engage in DTM, two of the children preformed LTM, however none preformed UTM throughout the assessment. Future research should investigate whether tongue lateralization requires prerequisistes such as DTM, LTM, and UTM. If that was the case, exercises to promote these movements would help children develop togue lateralization.

## Conclusion

The present study provides a comprehensive protocol to enable clinicians to assess the level of chewing, its components, and the prerequisites movements in children with DS and to identify the most appropriate starting point for interventions targeting chewing. Although tongue lateralization has obtained very limited attention in chewing research to date, the current findings highlight the importance of this skill in the development of rotary chewing, in addition to bite down and chew. The current findings indicate that an intervention to establish and reach fluency in all the chewing components may be necessary prior to teach the chewing sequence with higher textured foods.
